# Lymphatic Contrast-enhanced Ultrasound as a Noninvasive Predictor of Sentinel Lymph Node Metastasis in Breast Cancer: A Prospective Diagnostic Study

**DOI:** 10.1245/s10434-025-17705-y

**Published:** 2025-06-23

**Authors:** Jundong Yao, Husha Li, Hailong Wang, Binbin Liu, Shuai Cui, Qifan Liu, Chang Chang, Miao Deng, Shengjiang Chen

**Affiliations:** 1 Department of Ultrasound, The First Affiliated Hospital, College of Clinical Medicine of Henan University of Science and Technology, Luoyang, China; 2 Department of Surgery, The First Affiliated Hospital, College of Clinical Medicine of Henan University of Science and Technology, Luoyang, China; 3 Department of Pathology, The First Affiliated Hospital, College of Clinical Medicine of Henan University of Science and Technology, Luoyang, China; 4 Department of Breast Surgery, The First Affiliated Hospital, College of Clinical Medicine of Henan University of Science and Technology, Luoyang, China

**Keywords:** Breast cancer, Sentinel lymph node biopsy, Contrast-enhanced ultrasound, Axillary staging, Radiation-free

## Abstract

**Background:**

Sentinel lymph node biopsy (SLNB) using radioisotopes/blue dye remains limited by radiation exposure and logistical constraints. This study prospectively evaluates lymphatic contrast-enhanced ultrasound (L-CEUS) for SLN mapping and metastasis detection in early-stage breast cancer.

**Methods:**

A total of 251 consecutive patients underwent L-CEUS-guided SLN biopsy. Enhancement patterns (Types I-V) and filling defects were correlated with histopathology. Diagnostic performance was compared to conventional ultrasound parameters and surgical SLNB (*n* = 102).

**Results:**

L-CEUS successfully localized SLNs in 98.4% (247/251) of cases. Using Types III-V enhancement as metastatic criteria, L-CEUS demonstrated 99.0% sensitivity and 88.1% specificity (AUC 0.935). Incorporating filling defects improved specificity to 95.4% (AUC 0.967). Cortical thickness (>3.0 mm) outperformed nodal short-axis in metastasis prediction (AUC 0.874 vs. 0.702, *p* < 0.001). Compared with blue dye, L-CEUS identified fewer SLNs/patient (3.11 ± 0.81 vs. 3.59 ± 1.2, *p* = 0.001) with shorter procedural time (4.09 ± 0.25 vs. 12.12 ± 2.75 min, *p* < 0.001). Eight false-negatives involved micro-metastases (*n* = 3) and skip lesions (*n* = 5).

**Conclusions:**

L-CEUS provides high diagnostic accuracy for SLN evaluation while eliminating radiation exposure. Its real-time imaging capability and rapid procedural time support integration into standard axillary staging protocols, particularly where radioisotopes are unavailable. Prospective validation of long-term outcomes is warranted.

**Supplementary Information:**

The online version contains supplementary material available at 10.1245/s10434-025-17705-y.

Breast cancer (BC) remains a major global health challenge, ranking as the second most prevalent malignancy among women worldwide in 2022 according to World Health Organization estimates.^1^ The pathological status of axillary lymph nodes serves as a critical prognostic indicator for BC patients.^2^ While axillary lymph node dissection has historically been the standard approach, sentinel lymph node biopsy (SLNB) has emerged as a less invasive alternative for early-stage disease, effectively reducing complications, such as lymphedema and sensory impairment.^3^ The sentinel lymph nodes (SLNs), defined as the primary lymphatic drainage site from the tumor, reflects the histopathological status of the entire regional nodal basin.^4^ Accurate SLNs evaluation is therefore pivotal for staging and therapeutic decision-making.

Current SLNB protocols predominantly utilize radioisotope tracers combined with blue dye. However, this dual-tracer method presents notable limitations: it requires a prolonged waiting period (typically 2–24 hours) postinjection for optimal radiotracer migration and entails nonnegligible ionizing radiation burdens to both patients and medical staff.^5,6^ In China, blue dye alone is frequently employed to circumvent radiation concerns, yet its inability to provide preoperative anatomical localization significantly restricts surgical planning precision.^7^ These challenges have spurred the exploration of alternative localization technologies.

Contrast-enhanced ultrasound (CEUS), particularly lymphatic CEUS (L-CEUS), has shown promise as a radiation-free approach for SLNs mapping.^8,9^ Growing evidence supports its safety and efficacy in SLNs identification, leading to its provisional inclusion in the 2022 Chinese Anti-Cancer Society guidelines for BC SLNB.^10–16^ Nevertheless, the current recommendation carries low-quality evidence owing to insufficient prospective validation and limited data on L-CEUS–guided percutaneous biopsy accuracy.^10–13^ Notably, fewer than 15% of supporting studies employ prospective designs, highlighting a critical gap in high-quality evidence.

To address this knowledge gap, this prospective study aims to evaluate the diagnostic performance of L-CEUS in guiding SLNs biopsy and detecting nodal metastases in early-stage BC patients, thereby contributing evidence to optimize clinical guidelines.

## Materials and Methods

### Study Design and Participants

**Fig. 1:**
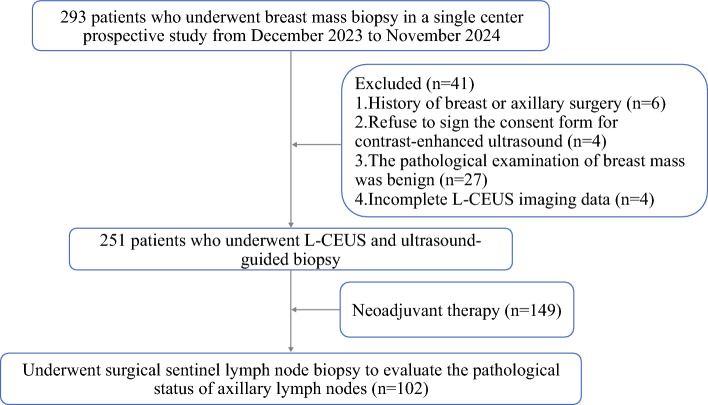
Study participants

This prospective diagnostic study consecutively enrolled patients undergoing breast mass aspiration at our tertiary medical center from December 2023 to November 2024. The inclusion criteria comprised 1) presence of lymph nodes requiring ultrasound-guided percutaneous biopsy based on suspicious ultrasound findings or clinical palpation abnormalities; 2) Completion of L-CEUS prior to biopsy; and 3) pathologically confirmed malignant breast lesions. Exclusion criteria included 1) history of prior surgery, radiotherapy, or chemotherapy involving the ipsilateral breast or axilla; 2) indeterminate pathological results from sentinel lymph node biopsy; 3) hypersensitivity to components of the ultrasound contrast agent; 4) incomplete L-CEUS imaging documentation; and 5) declined informed consent. The institutional review board approved the protocol (No. 2024-03-K0159) with written informed consent obtained from all participants. Figure 1 illustrates the participant flow diagram.

### Imaging Protocol

High-resolution ultrasonography was performed by using a premium-grade clinical system (Resona I9, Mindray) configured with dual transducers: a 14-MHz linear array for B-mode scanning and a 9-MHz curved array optimized for L-CEUS.

The ultrasound contrast agent used in this study consisted of sulfur hexafluoride microbubbles (SonoVue^®^, Bracco Imaging, Milan, Italy), prepared by reconstituting 59 mg of lyophilized powder with 5.0 mL of normal saline. Patients were positioned supine with the affected breast and axilla fully exposed. A standardized intradermal injection protocol was performed: 0.5 mL of the microbubble suspension was administered at 3, 6, 9, and 12 o’clock positions around the areolar margin (total 2.0 mL), followed by gentle manual massage of the injection area for 60 s to facilitate lymphatic drainage of the contrast agent.

Using the L9-3s transducer in contrast-enhanced ultrasound mode, SLNs were identified by tracking opacified lymphatic channels and detecting the first cluster of contrast-enhanced nodes along these pathways. Morphometric parameters including size, quantity, location, and enhancement patterns of SLNs were systematically documented. Subsequent B-mode evaluation with the L14-3Ws transducer was performed to characterize two-dimensional sonographic features of SLNs, with representative images archived for documentation.

### Image Interpretation

**Fig. 2:**
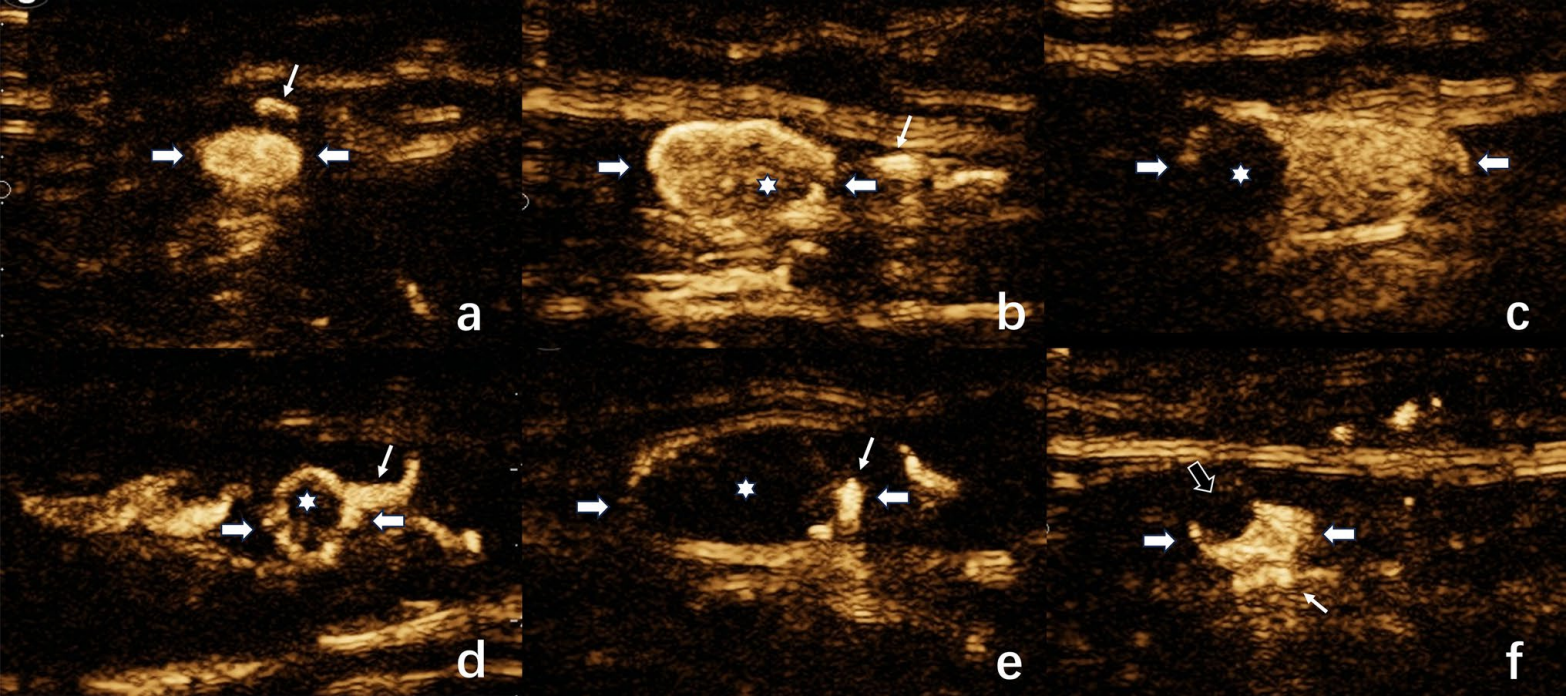
Enhancement characteristics of different types of SLNs on L-CEUS. **a** Type I: overall bright homogeneous enhancement throughout the SLNs (white thick arrow). **b** Type II: homogeneous enhancement of the cortex and uniform distribution of contrast agent (white asterisk) in the cortical area of lymph nodes. **c** Type III: uneven distribution of contrast agent in the cortical area of lymph nodes or the appearance of nonenhanced areas (white asterisk). **d** Type IV: peripheral complete or incomplete annular enhancement. **e** Type V: no contrast agent (white asterisk) enters the lymph node through the end of the enhanced afferent lymphatic vessels (white thin arrow). **f** The presence of filling defects (black arrow) in SLNs. Sentinel lymph nodes (white thick arrow), uneven enhancement or no enhancement (white asterisk), lymphatic vessels (white thin arrow), filling defects (black arrow). *SLNs* sentinel lymph nodes; *L-CEUS* lymphatic contrast-enhanced ultrasound

Two blinded CEUS-certified sonographers (H.L.W. and H.S.L., with 7 and 5 years of experience, respectively) conducted real-time consensus evaluations. A third independent reviewer (J.D.Y.) performed offline video analysis using predefined diagnostic criteria. Discordant cases were resolved through panel discussion. The enhancement patterns were classified as^10,15^:

- Type I: Homogeneous parenchymal enhancement (Fig. 2a)
- Type II: Uniform cortical enhancement (Fig. 2b)
- Type III: Heterogeneous cortical enhancement (Fig. 2c)
- Type IV: Complete/incomplete peripheral rim enhancement (Fig. 2d)
- Type V: Nonenhancement with lymphatic cutoff sign (Fig. 2e)

Types I-II were considered negative for metastasis, whereas Types III-V indicated positive status. Additional evaluation included assessment of subcapsular filling defects (Fig. 2f).

### Biopsy and Surgical Procedures

All visualized SLNs require ultrasound-guided percutaneous biopsy for definitive pathological confirmation. When multiple SLNs are identified, priority should be given to nodes exhibiting the highest enhancement category (particularly those suspicious for malignancy). In cases where all SLNs demonstrate identical enhancement characteristics, the first visualized node is recommended for initial biopsy. Ultrasound-guided core needle biopsy was performed using 18G coaxial needles.

Intraoperative SLNs mapping employed 0.1 mL of methylene blue injection at the superolateral peri-areolar region postanesthesia. All blue-stained nodes were surgically excised for histopathological examination, which served as the diagnostic reference standard.

### Statistical Analysis

Data analysis was conducted by using SPSS 26.0 (IBM Corp.). Continuous variables were expressed as mean ± SD with between-group comparisons using independent *t*-tests. Categorical variables were presented as frequencies (%) analyzed by χ^2^ or Fisher’s exact tests. Diagnostic accuracy parameters (sensitivity, specificity) were calculated against histopathology. A two-tailed *p*-value <0.05 defined statistical significance.

## Results

### Participant Characteristics

The study cohort comprised 251 consecutive female patients (median age, 55 years, range, 25–88) with histologically confirmed breast malignancies. Including invasive ductal carcinoma (*n* = 180), invasive lobular carcinoma (*n* = 37), invasive papillary carcinoma (*n* = 13), intraductal carcinoma (*n* = 11), ductal carcinoma in situ (*n* = 6), and invasive mucinous carcinoma (*n* = 4).

Successful SLNs localization was achieved in 98.4% (247/251) of cases following initial L-CEUS injection, with no procedure-related adverse events recorded. Among 251 L-CEUS-guided biopsy specimens, histopathology confirmed 100 metastatic and 151 benign SLNs. Of the total cohort, 102 patients underwent subsequent SLNB following neoadjuvant therapy exclusion, while 149 received neoadjuvant treatment (Table 1).

**Table 1:** Clinical characteristics of the initial population

Characteristics	Mean ± SD/no. patients (%)
Age (yr)	55.49±12.49
Maximum diameter of breast tumor (mm)	19.64±11.41
*Histology*	
Invasive ductal carcinoma	180 (71.7%)
Invasive lobular carcinoma	37 (14.7%)
Invasive papillary carcinoma	13 (5.2%)
Intraductal carcinoma	11 (4.4%)
Ductal carcinoma in situ	6 (2.4%)
Invasive mucinous carcinoma	4 (1.6%)
*ER/PR status*	
ER positive and/or PR positive	181 (72.1%)
ER and PR negative	70 (27.9%)
*Her2 status*	
Positive	149 (59.4%)
Negative	102 (40.6%)
*Clinical nodal status*	
Sonographically suspicious lymph nodes	205 (81.7%)
Clinically palpable suspicious lymph nodes	46 (18.3%)
*Pathological SLNs status*	
Positive	100 (39.8%)
Negative	151 (60.2%)

Continuous variables are presented as means ± SD or median with interquartile range in parentheses. *SLNs* sentinel lymph nodes; *SD* standard deviation; *ER* estrogen receptor; *PR* progesterone receptor

### Diagnostic Performance of L-CEUS

The evaluation results of the enhancement patterns by the two doctors reached a high inter-rater agreement (Kappa = 0.874). The enhancement pattern classification demonstrated strong correlation with metastatic status (Table 2): Type I: 100% benign (83/83); Type II: 97.9% benign (46/47); Type III: 71.2% metastatic (47/66); Type IV/V: 91.7–95.3% metastatic.

**Table 2:** Correlation between L-CEUS characteristics of SLNs and pathological results

	SLNs L-CEUS characteristics type					Total
	I	II	III	IV	V	
Pathology (-)	83	46	19	1	2	151
Pathology (+)	0	1	47	11	41	100

*SLNs* sentinel lymph nodes; *L-CEUS* lymphatic contrast-enhanced ultrasound; (−) negative; (+) positive

**Fig. 3:**
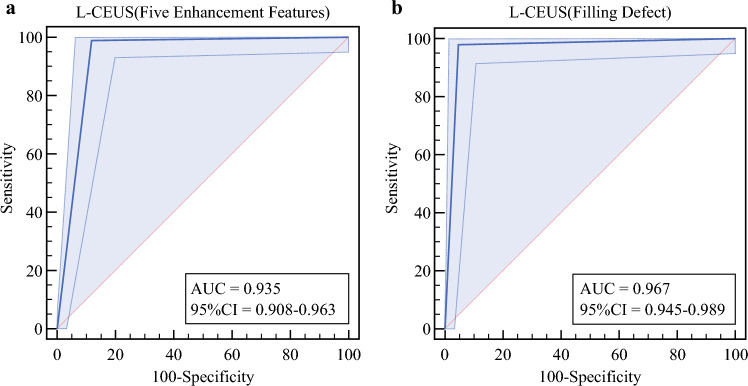
ROC curve for L-CEUS (5 enhancement patterns) **a** and L-CEUS (filling defect) **b**. *ROC* receiver operating characteristic; *L-CEUS* lymphatic contrast-enhanced ultrasound

Using Types III-V as metastatic criteria, L-CEUS achieved exceptional diagnostic accuracy: AUC 0.935 (95% confidence interval (CI) 0.908–0.963), sensitivity 99.0%, specificity 88.1% (Fig. 3a; Table 3). The single false-negative case in type II showed micrometastasis (<2 mm) on histology.

**Table 3:** Diagnostic efficacy of L-CEUS and morphological parameters for SLNs metastasis

	Cutoff value (mm)	AUC (95% CI)	Sen	Spe	Youden index
Five enhancement patterns	N/A	0.935 (0.908–0.963)	0.990	0.881	0.871
Filling defects	N/A	0.967 (0.945–0.989)	0.980	0.954	0.934
Short-axis diameter	6.5	0.702 (0.636–0.769)	0.790	0.556	0.346
Cortical thickness	3.0	0.874 (0.829–0.92)	0.880	0.729	0.609

*SLNs* sentinel lymph nodes; *L-CEUS* lymphatic contrast-enhanced ultrasound; *AUC* area under the curve; *CI* confidence interval; *Sen* sensitivity; *Spe* specificity

When incorporating filling defects as diagnostic criterion, specificity improved to 95.4% with maintained high sensitivity (98.0%), yielding an AUC of 0.967 (95% CI 0.945–0.989) (Fig. 3b; Table 3). False-positive cases (*n* = 7) were attributed to benign histological patterns including of which there were lymphoid follicular hyperplasia (*n* = 1), fat deposition (*n* = 2), and fusion of lymph nodes (*n* = 4).

### Conventional Ultrasound Parameters

Short-axis diameter and cortical thickness were significant difference between metastatic and benign SLNs (*p* < 0.05) (Table S3).

Both SLNs short-axis diameter (>6.5 mm) and cortical thickness (>3.0 mm) showed significant discriminative capacity (*p* < 0.001): Short-axis diameter: AUC 0.702 (95% CI 0.636–0.769), Youden index 0.346; Cortical thickness: AUC 0.874 (95% CI 0.829–0.92), Youden index 0.609; Cortical thickness demonstrated superior diagnostic performance compared to nodal short-axis (Table 3).

### Comparative Analysis with Surgical Blue Dye SLNB

In 102 surgical patients, L-CEUS identified fewer SLNs per patient than blue dye mapping (3.11 ± 0.81 vs. 3.59 ± 1.2, *p* = 0.001), with corresponding shorter procedural duration (4.09 ± 0.25 vs. 12.12 ± 2.75 min, *p* < 0.001) (Table 4). Eight false-negative cases were observed in L-CEUS, comprising micrometastases (*n* = 3) and skip metastases (*n* = 5) confirmed by SLNB histology (Table 5).

**Table 4:** Comparison of SLNs recognized by L-CEUS and blue dye

Parameter (mean ± SD)	L-CEUS (n = 102)	Blue dye (n = 102)	*p*
No. SLNs identified	3.11 ± 0.81	3.59 ± 1.2	0.001
Operating time	4.09 ± 0.25	12.12 ± 2.75	0.000

*SLNs* sentinel lymph nodes; *L-CEUS* lymphatic contrast-enhanced ultrasound; *SD* standard deviation

**Table 5:** Comparative analysis of L-CEUS and surgical SLNB

	Surgical SLNB		Total
	(−)	(+)	
L-CEUS (−)	93	8	101
L-CEUS (+)	0	1	1

*SLNs* sentinel lymph nodes; *L-CEUS* lymphatic contrast-enhanced ultrasound; *SLNB* sentinel lymph node biopsy; (−) negative; (+) positive

## Discussion

This prospective study establishes L-CEUS as a robust, radiation-free modality for preoperative SLNs evaluation in early-stage breast cancer, addressing critical limitations of conventional techniques while validating its clinical utility. By demonstrating high diagnostic accuracy (sensitivity 98.0%, specificity 95.4%) in a prospective cohort of 251 patients, our findings directly respond to the evidence gap highlighted in the Chinese Anti-Cancer Society’s provisional guidelines.^16^ The strong interrater agreement (*κ* = 0.874) in classifying L-CEUS enhancement patterns (Types I–V) further resolves reproducibility concerns raised in prior smaller studies, supporting standardized adoption across institutions.^10,12,14,17–20^

Conventional SLNB methods, reliant on radioisotopes or blue dye, are constrained by radiation exposure, prolonged procedural timelines, and limited preoperative anatomical visualization.^21,22^ L-CEUS circumvents these challenges through real-time anatomical guidance, significantly reduced procedural time compared with blue dye (4.09 vs. 12.12 min, *p* < 0.001), and elimination of radiation risks—features particularly advantageous in resource-limited settings.^19,20^ The malignancy gradient observed across L-CEUS patterns (0% in Type I to 95.3% in Type V) aligns with pathophysiological principles, where disrupted contrast perfusion reflects early metastatic infiltration. This stratification capability enhances preoperative decision-making, enabling tailored surgical planning.

The incremental cost of L-CEUS (approximately $70 per contrast dose in China) must be contextualized against its clinical benefits. In our cohort, all 251 patients underwent L-CEUS without contrast-related complications, reinforcing its safety.^23^ While conventional ultrasound avoids contrast costs, its higher false-negative rates (21% for short-axis diameter, 12% for cortical thickness) risk understaging—a critical concern given the prognostic implications of missed micrometastases.^24^ Cost-effectiveness analyses suggest L-CEUS may be justified in settings where radioisotopes are unavailable, as its 98.0% sensitivity could reduce downstream expenses from delayed axillary lymph node dissection or recurrence management.

For low-risk patients (cortical thickness ≤ 3 mm and short-axis diameter ≤ 6.5 mm), L-CEUS may be deferred in favor of direct SLNB or clinical surveillance. When L-CEUS demonstrates benign enhancement patterns (Type I-II), the exceptionally low false-negative rate (1%, 1/100 metastatic cases) supports consideration for SLNB omission. Conversely, high-risk patients (cortical thickness >3 mm or short-axis diameter >6.5 mm) require mandatory L-CEUS for metastatic confirmation. However, due to the predominance of skip metastases among false-negative cases (5/8), SLNB validation remains critical even with negative L-CEUS findings. This risk-adapted algorithm prioritizes diagnostic precision while minimizing unnecessary interventions, leveraging L-CEUS’s superior specificity (95.4%) to refine staging in clinically ambiguous cases.

While cortical thickness—a conventional ultrasound parameter—showed moderate diagnostic value (AUC = 0.874), L-CEUS achieved superior performance (AUC = 0.967), underscoring its ability to detect microstructural changes often missed by morphology-based criteria.^18,19^ The 7.9% false-negative rate (8/101 metastatic cases) highlights persistent challenges in identifying micro-metastases (<2 mm) and skip metastases, likely due to minimal tracer uptake in early lesions or atypical drainage pathways. These limitations necessitate cautious interpretation of negative L-CEUS results in high-risk patients, emphasizing that SLNB confirmation remains critical in such scenarios.

Notably, L-CEUS identified fewer SLNs per patient than blue dye (3.11 vs. 3.59, *p* = 0.001), potentially reflecting selective visualization of high-risk nodes. While this selectivity may reduce unnecessary biopsies of benign nodes, it raises concerns about missing non-enhancing metastatic foci in complex lymphatic networks. Thus, L-CEUS is optimally positioned for preoperative risk stratification in early-stage tumors, whereas advanced cases may benefit from hybrid approaches combining L-CEUS with dye-guided SLNB to optimize detection.^25,26^

Despite its strengths, this study has limitations. The single-center design and exclusion of postneoadjuvant therapy patients (*n* = 149) limit generalizability to treatment-modified axillary basins. Future research should prioritize three objectives: 1) validating L-CEUS in post-neoadjuvant cohorts to assess its role in treatment response evaluation; 2) standardizing protocols for integrating L-CEUS with existing SLNB techniques; and 3) conducting cost-effectiveness analyses to guide implementation across diverse healthcare systems.

## Conclusions

This study supports L-CEUS as a valuable tool for preoperative SLN evaluation in early-stage breast cancer, offering radiation-free operation, real-time guidance, and high diagnostic accuracy. Its ability to stratify metastatic risk through enhancement patterns (0% malignancy in Type I to 95.3% in Type V) provides actionable insights for surgical planning. While the 7.9% false-negative rate underscores the need for intraoperative SLNB confirmation in high-risk cases, the exceptionally low false-negative rate (1%) in L-CEUS-negative, low-risk patients suggests potential for SLNB omission in select cohorts. The incremental cost of L-CEUS (approximately $70 per dose) is offset by its capacity to reduce understaging risks and downstream treatment costs, particularly in regions lacking radioisotope access. These findings advocate for broader integration of L-CEUS into clinical guidelines, particularly in settings prioritizing minimally invasive staging. Future efforts should refine multimodal protocols to balance precision, cost, and accessibility in axillary management.

## Supplementary Information

Below is the link to the electronic supplementary material.

Supplementary file1 (DOCX 22 KB)
