# Supplementary material for: Lymphatic Contrast-enhanced Ultrasound as a Noninvasive Predictor of Sentinel Lymph Node Metastasis in Breast Cancer: A Prospective Diagnostic Study
Source: Ann Surg Oncol. 2025 Jun 23;32(12):8693–700. doi: 10.1245/s10434-025-17705-y (PMC12534311; doi:10.1245/s10434-025-17705-y)
Supplement: Supplementary file 1 — Supplementary file1 (DOCX 22 KB) [file 10434_2025_17705_MOESM1_ESM.docx]

**Table S1** The Kappa value of the enhancement patterns by the two doctors

| **Doctor** |  | **Doctor 1** | | | | |  |
| --- | --- | --- | --- | --- | --- | --- | --- |
|  | **Patterns** | **I** | **IIa** | **IIb** | **III** | **IV** | **Total** |
| **Doctor 2** | **I** | 74 | 5 | 0 | 0 | 0 | 79 |
|  | **IIa** | 4 | 38 | 7 | 0 | 0 | 49 |
|  | **IIb** | 0 | 8 | 60 | 0 | 0 | 68 |
|  | **III** | 0 | 0 | 0 | 12 | 0 | 12 |
|  | **IV** | 0 | 0 | 0 | 0 | 43 | 43 |
|  | **Total** | 78 | 51 | 67 | 12 | 43 | 251 |

**Table S2** Correlation between filling defects of L-CEUS and pathological results

|  | **Filling defects of L-CEUS** | | Total |
| --- | --- | --- | --- |
|  | (-) | (+) |  |
| Pathology (-) | 144 | 7 | 151 |
| Pathology (+) | 2 | 98 | 100 |

Note. L-CEUS: lymphatic contrast-enhanced ultrasound, (-): negative, (+): positive.

**Table S3** Comparison of morphological parameters between metastatic and benign SLNs

| Parameter (Mean±SD) | Benign SLNs  (n=151) | Metastatic SLNs  (n=100) | *p* value |
| --- | --- | --- | --- |
| Long diameter | 17.70±7.095 | 17.86±7.587 | 0.870 |
| Width diameter | 7.395±6.766 | 9.184±3.726 | 0.017 |
| Cortical thickness | 2.393±1.925 | 6.933±4.275 | 0.000 |

Note. SLNs: sentinel lymph nodes, SD: standard deviation.
